# Oyster‐derived dipeptides RI, IR, and VR promote testosterone synthesis by reducing oxidative stress in TM3 cells

**DOI:** 10.1002/fsn3.3589

**Published:** 2023-08-06

**Authors:** Yongqiu Yan, Mingliang Li, Ying Wei, Fuhuai Jia, Yanying Zheng, Gang Tao, Feifei Xiong

**Affiliations:** ^1^ College of Biosystems Engineering and Food Science Zhejiang University Hangzhou China; ^2^ Ningbo Yufangtang Biotechnology Co., Ltd. Ningbo China; ^3^ Ningbo Yuyi Biotechnology Co., Ltd. Ningbo China; ^4^ School of Food Science and Technology Jiangnan University Wuxi China; ^5^ Department of Food Science and Engineering Beijing University of Agriculture Beijing China

**Keywords:** antioxidant, dipeptide, ROS, testosterone

## Abstract

Short peptides have gained widespread utilization as functional constituents in the development of functional foods due to their remarkable biological activity. Previous investigations have established the positive influence of oysters on testosterone biosynthesis, although the underlying mechanism remains elusive. This study aims to assess the impact of three dipeptides derived from oysters on the oxidative stress state of TM3 cells induced by AAPH while concurrently examining alterations in cellular testosterone biosynthesis capacity. The investigation encompasses an analysis of reactive oxygen species (ROS) content, antioxidant enzyme activity, apoptotic status, and expression levels of crucial enzymes involved in the testosterone synthesis pathway within TM3 cells, thus evaluating the physiological activity of the three dipeptides. Additionally, molecular docking was employed to investigate the inhibitory activity of the three dipeptides against ACE. The outcomes of this study imply that the oxidative stress state of cells impedes the synthesis of testosterone by inhibiting the expression of essential proteins in the testosterone synthesis pathway. These three dipeptides derived from oysters ameliorate cellular oxidative stress by directly scavenging excess ROS or reducing ROS production rather than enhancing cellular antioxidant capacity through modulation of antioxidant enzyme activity. These findings introduce a novel avenue for developing and utilizing antioxidant peptides derived from food sources.

## Graphical abstract

This study reports on three oyster‐derived dipeptides with antioxidant activity, RI, IR, and VR. The results of this study suggest that these dipeptides may promote testosterone biosynthesis by alleviating oxidative stress in TM3 cells, thereby increasing the expression of critical enzymes in the testosterone synthesis pathway. In addition, these three dipeptides may alleviate the oxidative stress state of cells by directly scavenging excess ROS from cells rather than increasing the activity of antioxidant enzymes in cells.

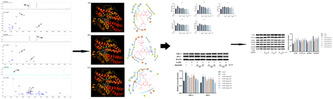

## 1 INTRODUCTION

Testosterone is an important androgen synthesized mainly in the Leydig cells of the testis (Nwachukwu & Aluko, 2019), with cholesterol as the initial substrate (Schiffer et al., 2018). Cholesterol transport across the mitochondrial membrane by the StAR protein is the rate‐limiting step in testosterone synthesis. The CYP11A1 mediates the conversion of cholesterol to pregnenolone in the inner mitochondrial membrane, pregnenolone is converted to progesterone by 3β‐HSD (Zhao et al., 2016), and androstenedione is catalyzed by 17β‐HSD to produce the final product testosterone (Han et al., 2018). Testosterone plays a crucial role in male fertility by maintaining sperm production and maturation. Decreased testosterone levels can disrupt spermatogenesis and lead to immature sperm (Barati et al., 2020). Testosterone deficiency also can lead to reduced muscle mass, erectile dysfunction, increased fatigue, hypogonadism, low fertility, depressed mood, poor memory, and sleep problems (Li, Zhou, Wei, et al., 2020; Tsametis & Isidori, 2018).

The impact of reactive oxygen species (ROS) on male fertility is of particular concern (Aitken, 2020), as oxidative stress resulting from an imbalance between ROS and antioxidants is considered a significant contributor to male infertility in 30%–80% of men (Ali et al., 2021). ROS are involved in various pathological conditions in the testis and are essential in inhibiting testosterone synthesis (Begum et al., 2018). Excessive ROS induces lipid peroxidation in the reproductive system and disrupts DNA and protein functions in sperm or testicular cells (Darbandi et al., 2018). ROS have been reported to inhibit testosterone production by suppressing steroidogenic enzyme gene expression through c‐Jun‐mediated inhibition of Nur77 trans‐activation (Baskaran et al., 2021). The leading causes of increased ROS include aging, heat stress, smoking, alcohol consumption, and radiation (Barati et al., 2020). In addition, the catalytic action of cytochrome P450 enzymes in steroid‐producing cells also leads to extraordinarily high levels of ROS production, which can inhibit steroid hormone synthesis at the level of cholesterol transfer (Greifova et al., 2020). Maintaining a balanced redox status is essential for the normal functioning of the male reproductive system (Baskaran et al., 2021).

Food‐derived bioactive peptides are small‐molecule peptide sequences obtained from food proteins by enzymatic hydrolysis or fermentation. These peptides exhibit superior physiological activities compared to their parent proteins, such as antihypertensive, antimicrobial, immunomodulatory, and antioxidant (Chai et al., 2020; Chakrabarti et al., 2018; Chalamaiah et al., 2018). The activity of these peptides is usually influenced by the protein source, amino acid composition and arrangement, molecular weight, and other factors (Peighambardoust et al., 2021). The human body can directly absorb the dipeptides through the intestinal wall through the H^+^/peptide cotransporter PepT1 (Miner‐Williams et al., 2014). Therefore, dipeptide has a good anti‐digestion ability and is more likely to have physiological activity. Physiological activities of dipeptides have been widely reported, such as the antioxidant activity of L‐carnosine (Cao et al., 2021), the protective effect of dipeptide YA against alcoholic liver injury (Siregar et al., 2020), the enhancement of dopamine activity by dipeptide WY to improve memory (Ano et al., 2019), the control of neuronal activity of energy metabolism by dipeptide GG (Shiraishi et al., 2020), the antidepressant activity of dipeptide YL (Mizushige et al., 2020), and so on.

Numerous studies have highlighted the diverse physiological activities of oyster peptides, including their antioxidant, immunomodulatory, antibacterial, anti‐inflammatory, anti‐fatigue, anti‐melanogenic, and pro‐male reproductive effects (Hao et al., 2021; Ulagesan et al., 2022). However, most of these studies have focused on oyster peptide mixtures, and limited information is available regarding the physiological activities of specific oyster peptide fractions. This study aims to investigate the physiological activities of three major oyster dipeptides, namely RI (Arg‐Ile), IR (Ile‐Arg), and VR (Val‐Arg). To establish a model of oxidative stress, 2,2′‐azo(2‐amidinopropane) hydrochloride (AAPH) was employed in TM3 cells. The effects of these three dipeptides on cellular testosterone synthesis capacity under oxidative stress conditions were examined by evaluating a series of indexes. By elucidating the beneficial effects of these dipeptides, this study aims to shed light on the intervention strategies that can enhance cellular testosterone synthesis capacity in the presence of oxidative stress.

## 2 MATERIALS AND METHODS

### 2.1 Materials

Peptides RI (Arg‐Ile), IR (Ile‐Arg), and VR (Val‐Arg) were synthesized by ChinaPeptides Co., Ltd. (Shanghai, China), with purity ≥99%. ROS Assay Kit, Annexin V‐FITC Apoptosis Detection Kit, Total Superoxide Dismutase Assay Kit, and Total Glutathione Peroxidase Assay Kit were purchased from Beyotime (Shanghai, China). Glutathione S‐transferase Assay Kit and Catalase Assay Kit were purchased from Jiancheng Bioengineering Institute (Nanjing, China). Mouse testosterone ELISA Kit was obtained from SenBeiJia Biological Technology Co., Ltd. (Nanjing, China). C‐Jun N‐terminal Kinase (JNK) antibody, Phospho‐JNK antibody, Nuclear respiratory factor‐2 (NRF‐2) antibody, Heme oxygenase‐1 (HO‐1) antibody, CYP11A1 antibody, and StAR antibody were purchased from Abcam (Shanghai, China). 17β‐HSD antibody was purchased from Proteintech Group, Inc. (Wuhan, China). 3β‐HSD antibody was obtained from Santa Cruz (Shanghai) Co., Ltd. (Shanghai, China). Cholecystokinin octapeptide (CCK‐8) was obtained from DOJINDO (Kyushu, Japan). AAPH was purchased from Sigma‐Aldrich (St. Louis, USA).

### 2.2 Cell culture

TM3 cells were purchased from the National Experimental Cell Resource Sharing Platform (Beijing, China). Cells were cultured in a constant temperature incubator (Thermo Fisher Scientific, Massachusetts, USA) with 5% CO_2_ at 37°C. Cell culture was performed in DMEM/F12 medium with 2.5% fetal bovine serum, 5% horse serum, and 1% penicillin–streptomycin solution.

### 2.3 Identification of oligopeptides

The oyster peptide mixture was obtained from oyster meat using a series of processing steps, including homogenization, proteolysis, centrifugation, ceramic membrane filtration, concentration, sterilization, and spray drying. The fractionations of the oligopeptide were detected with HPLC–MS/MS (8060, Shimadzu Corporation, Japanese) to reveal the primary structure. The fractionations were subjected to a Q3‐Scan with a mass scan range of m/z 132–600. The elution was conducted using mobile phase A, consisting of pure water (containing 0.1% formic acid), and mobile phase B consisting of 80% aqueous acetonitrile (containing 0.1% formic acid). At the same time, the flow rate was 0.2 mL/min. The gradient elution curves were 0–15 min, B:0%–50%; 15–20 min, B:50%–00%; 20–25 min, B:100%; and 25–35 min, B:0%. Then, the fractionations were subjected to a second scan with the collision energy set to −25 to identify the amino acid sequences of the peptides.

### 2.4 Cytotoxicity test

Cells were inoculated into 96‐well plates at a density of 1 × 10^5^/mL and incubated for 24 h. After incubation, the medium was discarded, and different concentrations of RI, IR, and VR (0, 10, 20, 30, 40, and 50 μg/mL) were added to the respective wells. The cells were then incubated for an additional 24 h, after which 10 μL of CCK‐8 was added to each well and mixed. After 1.5 h incubation, absorbance was measured at 450 nm to assess cell viability.

### 2.5 Modeling of cellular oxidative stress

Cells were inoculated into 96‐well plates at a density of 1 × 10^5^/mL and incubated for 24 h. Following this incubation period, the medium was removed, and various concentrations of AAPH (0, 200, 400, 600, and 800 μmol/L) were added to the wells. The cells were then incubated for an additional 24 h. Cell viability was assessed using the CCK‐8 assay. In a separate 96‐well black opaque plate, cells were incubated as described above with different concentrations of AAPH. After 24 h of incubation, 2′,7′‐dichlorofluorescein diacetate (DCFH‐DA) was added to each well. The cells were incubated for an additional 30 min and then washed three times with serum‐free medium. The fluorescence intensity of each well was detected using a fluorescent microplate reader to reflect the ROS content in the cells.

### 2.6 Dipeptide processing and cell sample collection

Cells were inoculated into 6‐well plates at a concentration of 1 × 10^5^/mL and incubated for 24 h. Following this incubation period, AAPH was added to the wells. Subsequently, RI, IR, and VR dipeptides were added to each well, and the cells were incubated for an additional 24 h. For ROS detection, cells were seeded into 96‐well black opaque plates at a concentration of 1 × 10^5^/mL and incubated for 24 h. AAPH was then added, followed by the addition of the dipeptides. After 24 h of incubation, the cells in the 96‐well black opaque plates were directly used for the ROS assay. For apoptotic status measurement, cells from the 6‐well plates were harvested. The cells were analyzed using a flow cytometer to determine their apoptotic status. Cellular proteins were collected for antioxidant enzyme viability assay by adding 300 μL of radioimmunoprecipitation assay (RIPA) lysate to each well of a 6‐well plate, and the lysate was centrifuged at 10,000 × *g* for 10 min. The cell culture supernatant in 6‐well plates was collected for testosterone content assay.

### 2.7 Western blot

Briefly, 300 μL of phenylmethanesulfonyl fluoride (PMSF) and RIPA lysis buffer (PMSF:RIPA = 1:100) were added to each well of the 6‐well plate, and the cells were centrifuged at 10,000 × *g* for 10 min after sufficient lysis. The cellular proteins were collected for protein concentration determination using the bicinchoninic acid (BCA) method. The cellular proteins were mixed with protein loading buffer and boiled for 5 min to denature the proteins completely. The proteins were subjected to sodium dodecyl sulfate‐polyacrylamide gel electrophoresis (SDS‐PAGE) with a sample quality of 20 μg. The protein bands were transferred to polyvinylidene difluoride (PVDF) membranes under constant current conditions. The PVDF membranes were blocked with 5% skimmed milk powder for 2 h and incubated with antibodies at 4°C overnight, followed by incubation with the appropriate secondary antibody for 2 h. Finally, the protein bands were stained by electrochemiluminescence (ECL) and analyzed using a chemiluminescence imaging system (Clinx Science, Shanghai, China). Optical density analysis was performed using ImageJ software (National Institutes of Health, Bethesda, MD, USA).

### 2.8 Molecular docking

Molecular docking was employed to investigate the impact of the amino acid composition and sequence of the peptides (RI, IR, and VR) on their physiological activity. Specifically, the binding sites and mechanism of action of these peptides to the ACE protein were predicted using molecular docking. The RCSB Protein Data Bank obtained the ACE protein structure, with the corresponding PDB ID being 1O86. The molecular docking analysis was conducted using Schrodinger software, taking into account the docking method described in the literature (Liu et al., 2021).

### 2.9 Statistical analysis

All data are from at least three independent replicate experiments. Data were statistically analyzed using SPSS 27 software, and images were generated using GraphPad Prism software. All data are expressed as mean ± SEM, and *p* < .05 was considered significantly different.

## 3 RESULTS

### 3.1 Peptide sequences

**FIGURE 1:**
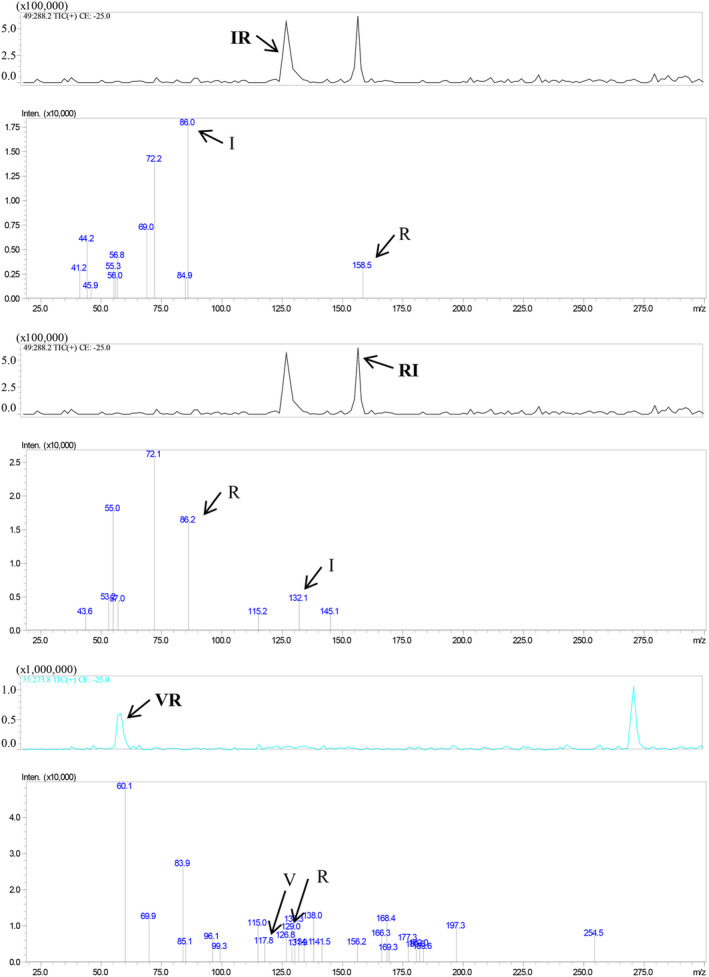
The results of peptide sequence detection.

Figure 1 displays the outcomes of the peptide sequence analysis. Three dipeptides, namely IR, RI, and VR, were identified from the oyster peptide mixture. Their identification was established by analyzing the parent molecule and fragment ion information and comparing them with oyster protein sequences in the NCBI database.

### 3.2 Cytotoxicity test results

**FIGURE 2:**
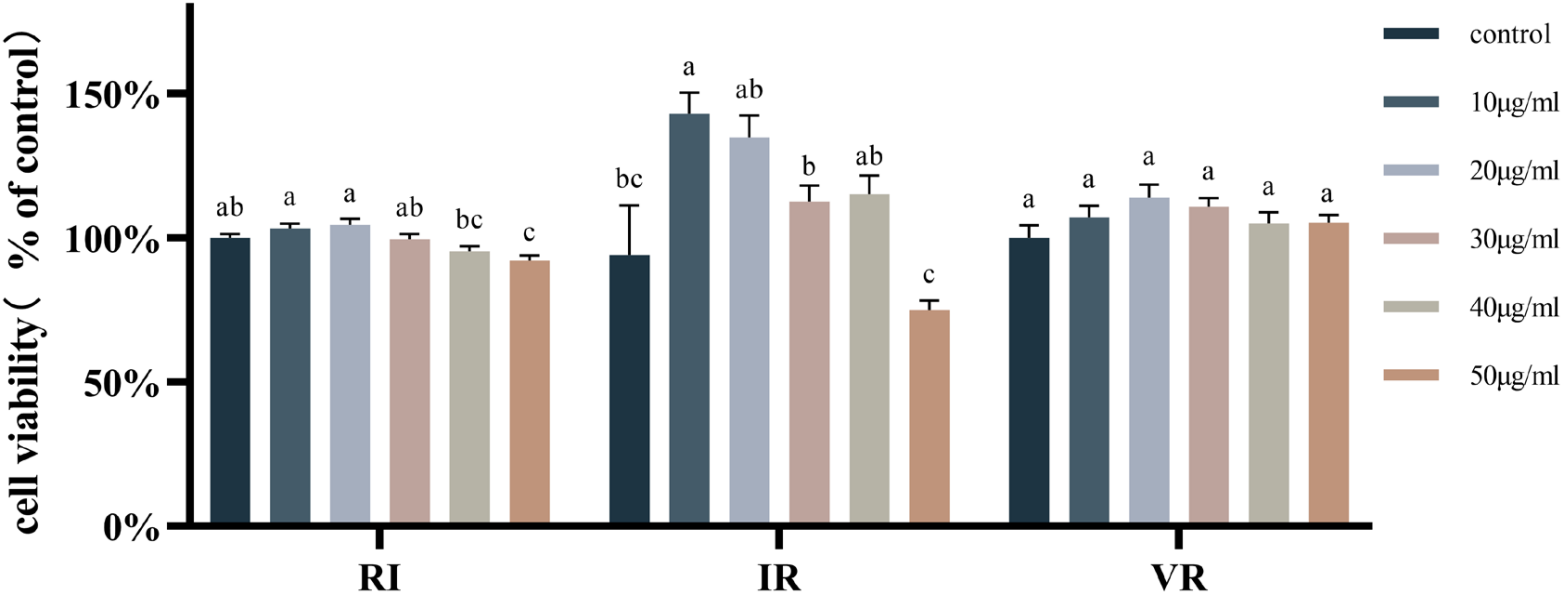
Cell viability after treatment with different concentrations of dipeptides, *n* = 10, different letters indicate significant differences.

Figure 2 demonstrates the impact of different peptide concentrations on cell viability. RI, at concentrations of 10, 20, and 50 μg/mL, and IR, at a concentration of 10 μg/mL, exhibited significant alterations in cell viability (*p* < .05). While VR, at various concentrations, induced changes in cell viability, the observed differences were not statistically significant (*p* > .05). Consequently, 20 μg/mL and 40 μg/mL concentrations were selected as the final peptide concentrations for subsequent treatments of TM3 cells in the subsequent experiments.

### 3.3 Oxidative stress modeling

**FIGURE 3:**
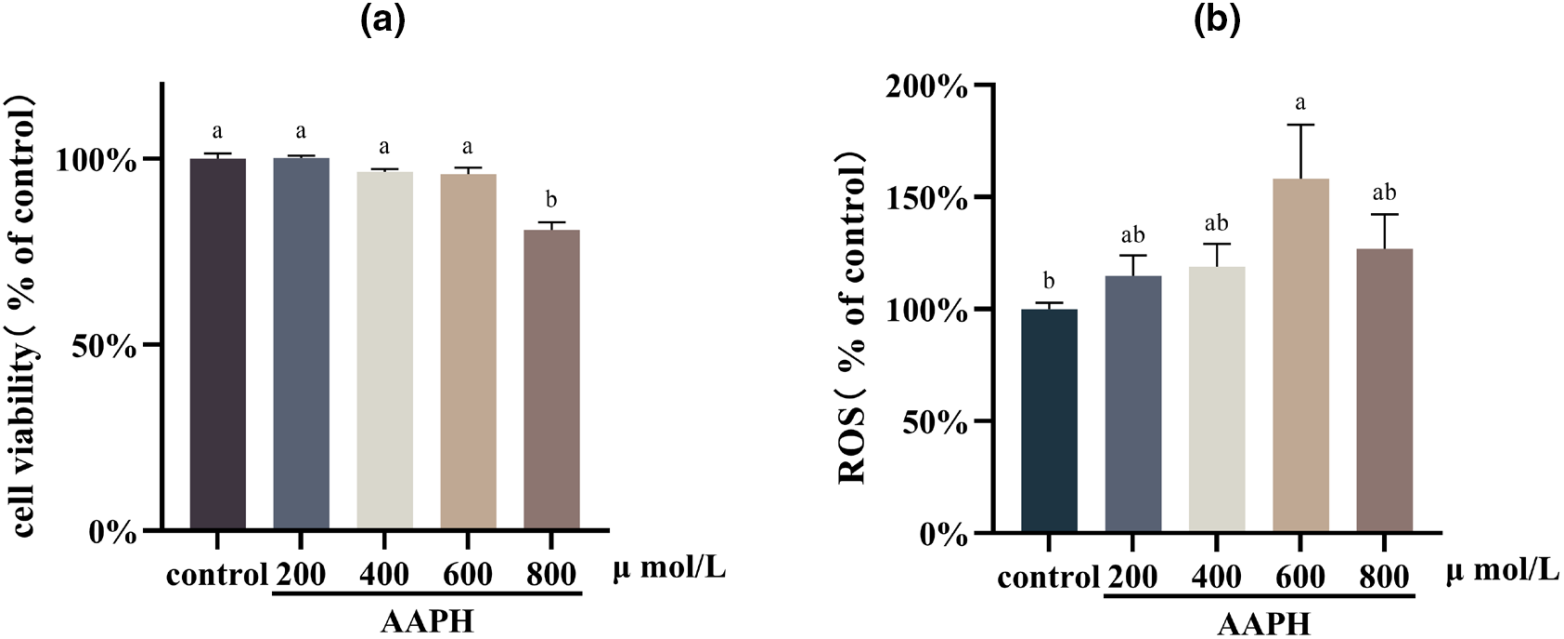
(a) Cell viability after treatment with different concentrations of AAPH, *n* = 10. (b) Relative intracellular ROS content after treatment with different concentrations of AAPH, *n* = 10. Different letters indicate significant differences.

In Figure 3a, it can be observed that AAPH at concentrations of 200, 400, and 600 μmol/L did not have a significant impact on cell viability after 24 h (*p* > .05). However, a concentration of 800 μmol/L of AAPH significantly reduced cell viability (*p* < .05).

Figure 3b displays the intracellular ROS content after different concentrations of AAPH treatments. Treatment with AAPH at a concentration of 600 μmol/L for 24 h resulted in a notable increase in intracellular ROS content (*p* < .05). Conversely, cells treated with AAPH at 800 μmol/L did not exhibit a significant increase in ROS content compared to the control group (*p* > .05). This observation could be attributed to the insufficient production of ROS caused by the significant decrease in cell viability. Consequently, a cellular oxidative stress model was established in this study by treating TM3 cells with 600 μmol/L of AAPH for 24 h.

### 3.4 Dipeptides improve the oxidative stress state of cells

**FIGURE 4:**
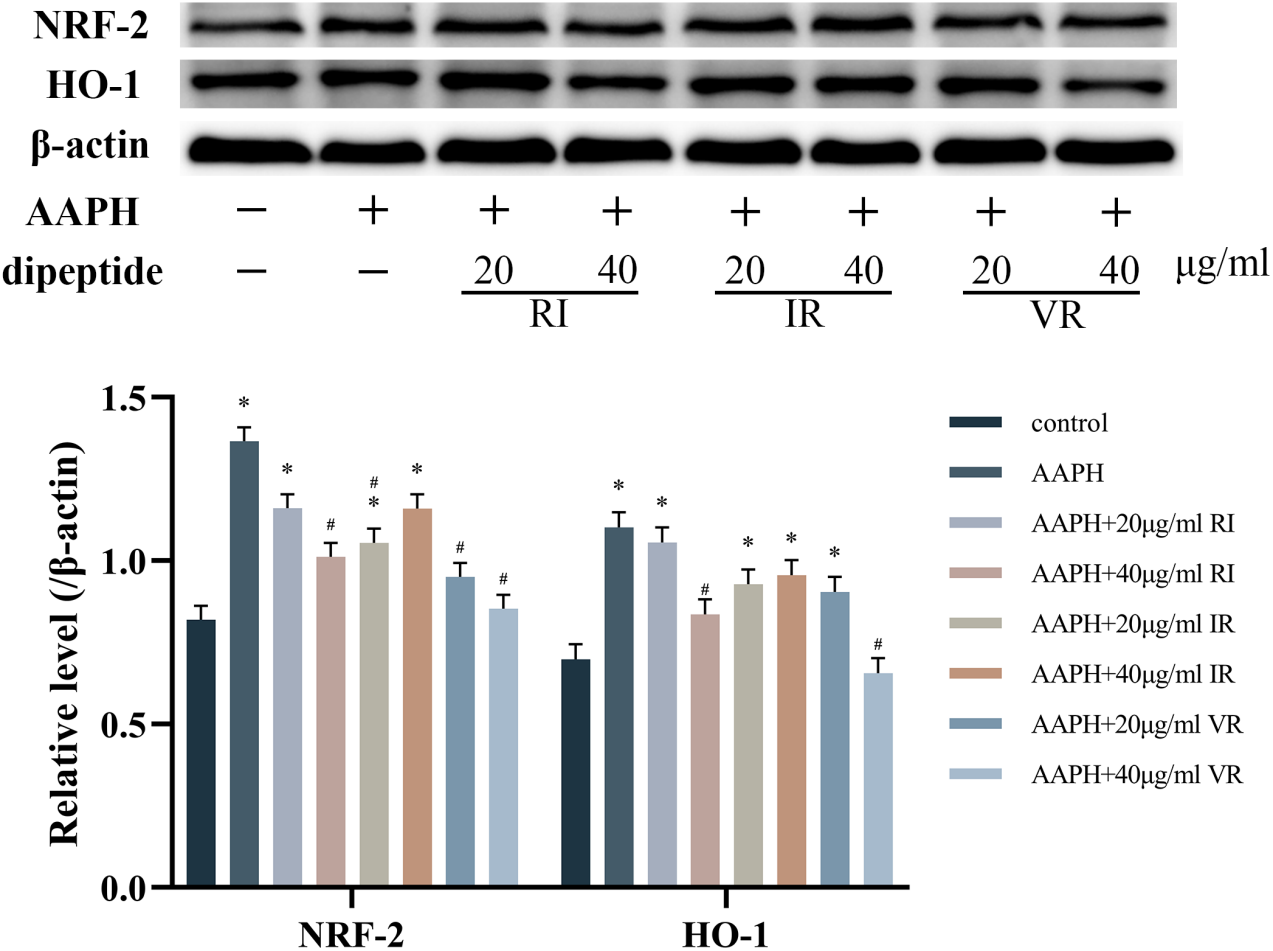
Western blot bands and grayscale analysis of NRF‐2 and HO‐1, *n* = 3, *significantly different compared to the control group, #significantly different compared to the AAPH group.

Figure 4 illustrates the impact of AAPH treatment on the expression levels of NRF‐2 and HO‐1 proteins in the cells. AAPH treatment significantly increased the expression levels of NRF‐2 and HO‐1 proteins (*p* < .05). However, after treatment with various concentrations of the three dipeptides, the expression levels of both proteins exhibited varying degrees of reduction. The effects of RI and VR were found to be dose‐dependent. Notably, the dipeptide VR substantially decreased the cells' expression levels of NRF‐2 and HO‐1 proteins. Specifically, treatment with 40 μg/mL of VR restored the expression levels of these two proteins to a level similar to that observed in the control group.

**FIGURE 5:**
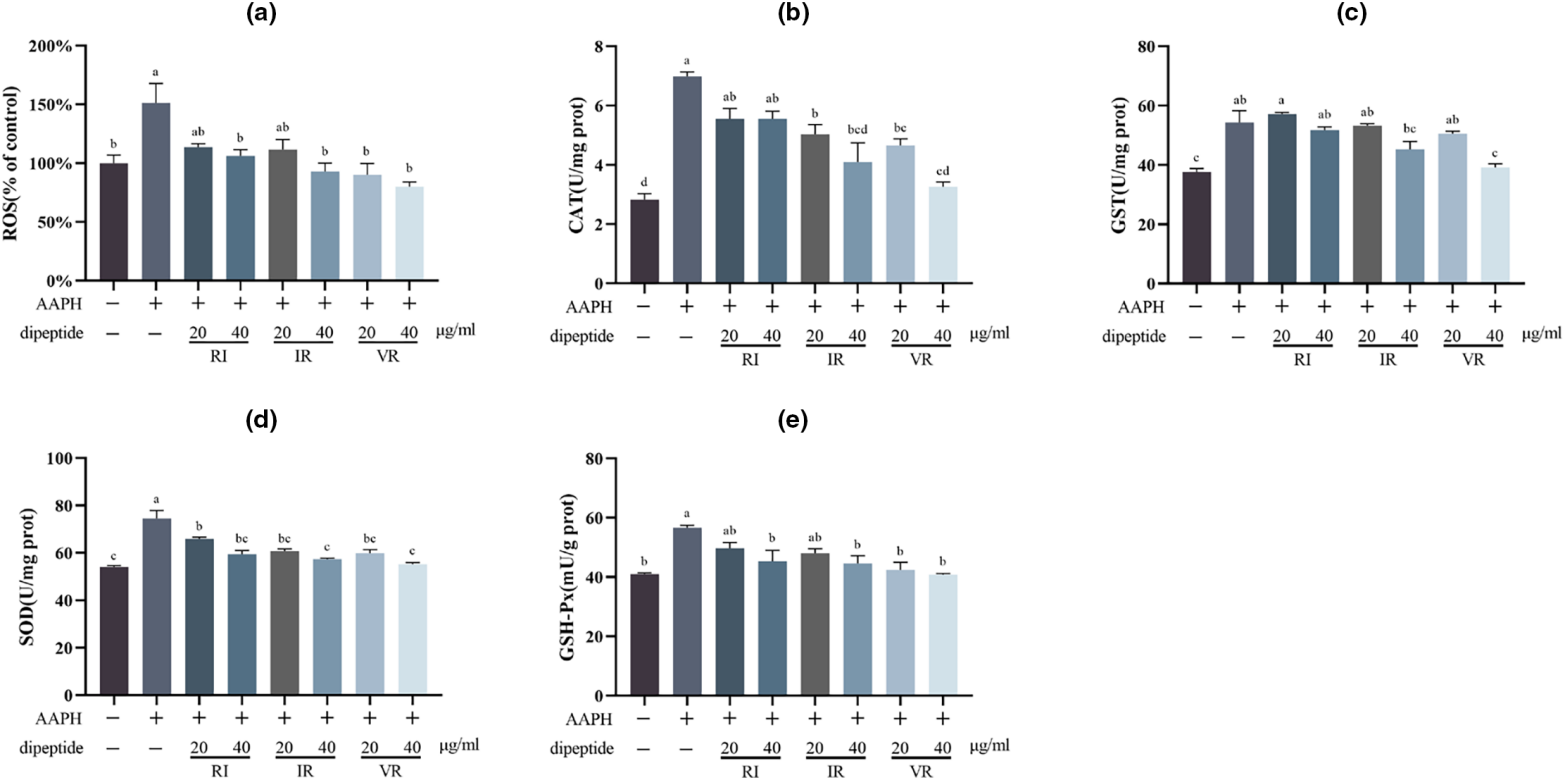
(a) The relative content of ROS in cells, *n* = 10. (b) Enzymatic activity of CAT, *n* = 3. (c) Enzymatic activity of GST, *n* = 3. (d) Enzymatic activity of SOD, *n* = 3. (e) Enzymatic activity of GSH‐Px, *n* = 3. Different letters indicate significant differences.

Figure 5a presents the results of the ROS assay, indicating a significant increase in ROS levels in cells following AAPH treatment (*p* < .05). These findings align with the NRF‐2 and HO‐1 assay results. Interventions with the three dipeptides led to varying degrees of reduction in cellular ROS levels, with significant differences (*p* < .05) observed in all treatment groups compared to the AAPH group, except for RI and IR at a concentration of 20 μg/mL.

Figure 5b–e demonstrates the activities of the antioxidant enzymes CAT, GST, SOD, and GSH‐Px in the cells. A significant increase (*p* < .05) in the activities of these enzymes was observed alongside the significant rise in ROS levels. The intervention of the three dipeptides at different concentrations resulted in varying degrees of reduction in antioxidant enzyme activity within the cells, indicating mitigation of oxidative stress. These findings are consistent with the results of the ROS content assay. Furthermore, this assay highlighted a dose‐dependent effect of the three dipeptides.

### 3.5 Dipeptides attenuate apoptosis

**FIGURE 6:**
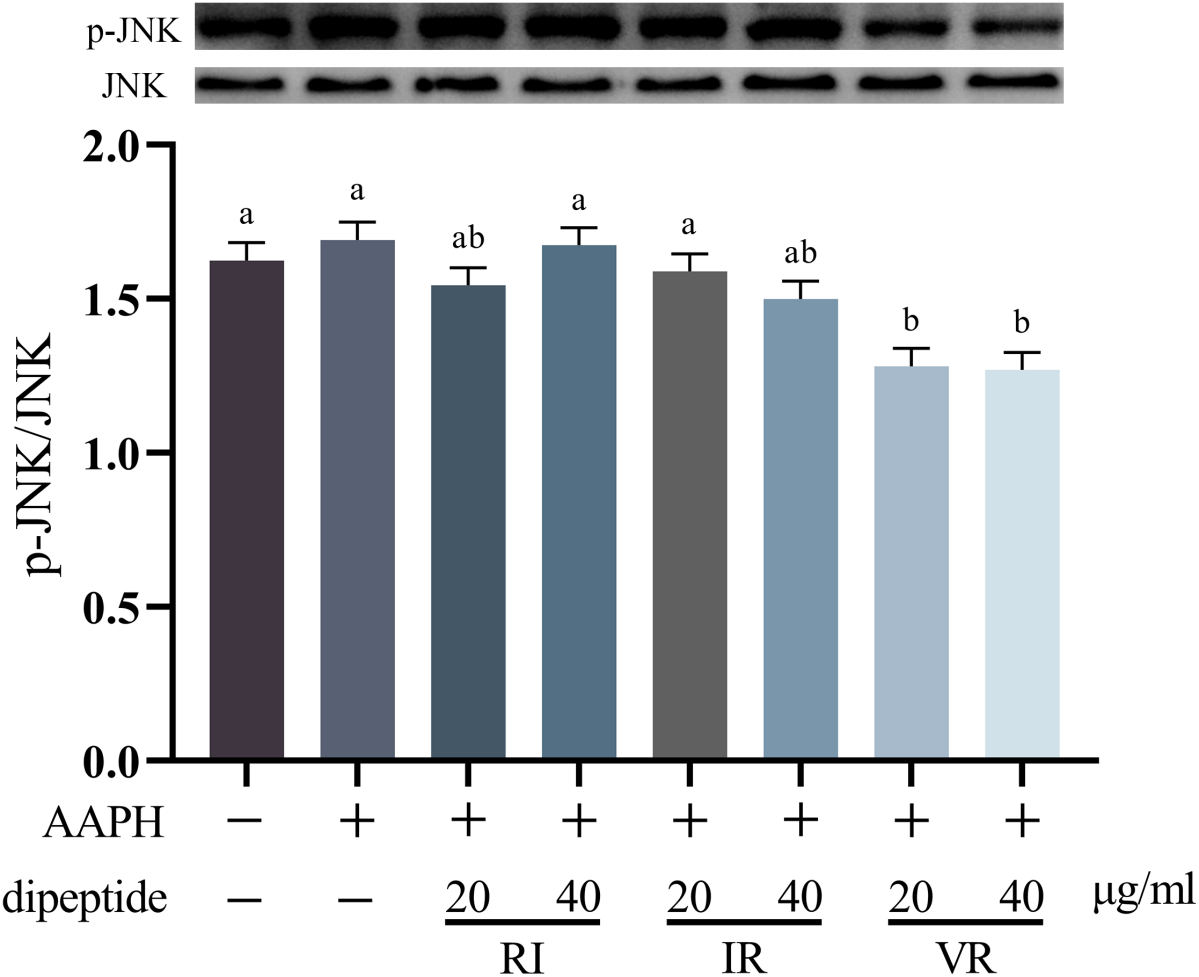
Western blot bands and grayscale analysis of p‐JNK and JNK, *n* = 3, different letters indicate significant differences.

Figure 6 displays the effect of AAPH treatment on the phosphorylation level of the JNK protein in the cells. Although treatment with AAPH increased JNK phosphorylation level, this increase did not reach statistical significance compared to the control group (*p* > .05). However, under dipeptide intervention, the phosphorylation level of the JNK protein was reduced to varying degrees compared to both the control and AAPH groups. Notably, the treatment groups with different concentrations of VR exhibited significant differences compared to the control and AAPH groups (*p* < .05).

**FIGURE 7:**
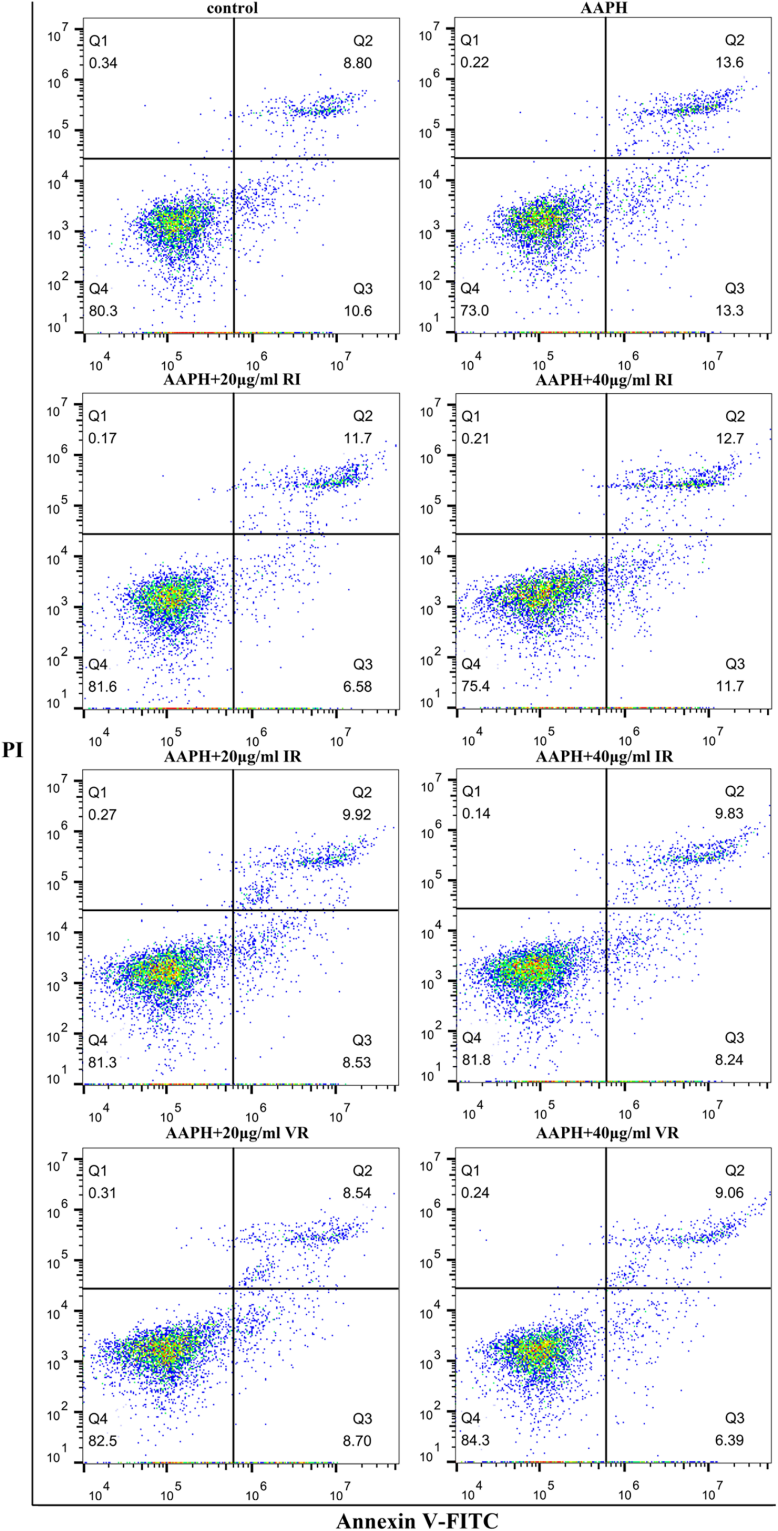
Apoptosis results were detected by flow cytometry. Upper right quadrant: necrotic cells. Lower left quadrant: live cells. Lower right quadrant: apoptotic cells.

Figure 7 presents the flow assay results of the cells, indicating that AAPH treatment significantly increased the percentage of apoptotic and necrotic cells by 7.5% compared to the control group. However, under dipeptide intervention, the proportion of apoptotic and necrotic cells exhibited varying degrees of reduction. Notably, the two treatment groups with VR showed the highest cell survival rates among all the interventions.

### 3.6 Expression of key enzymes in the testosterone synthesis pathway and the synthesis of testosterone

**FIGURE 8:**
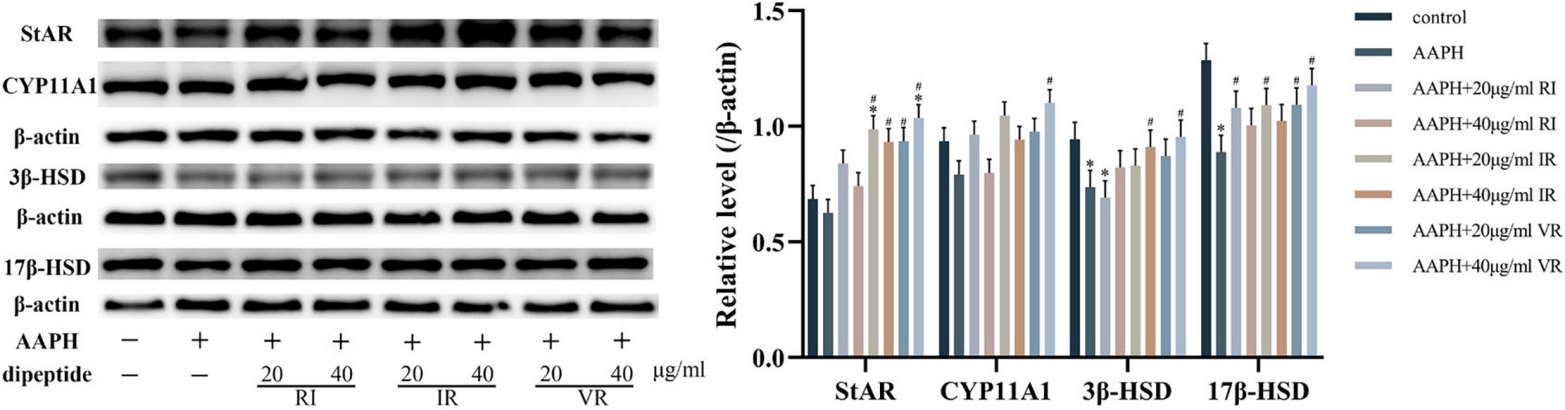
Western blot bands and grayscale analysis of StAR, CYP11A1, 3β‐HSD, and 17β‐HSD, *n* = 3, *significantly different compared to the control group, #significantly different compared to the AAPH group.

Figure 8 demonstrates the protein expression levels of StAR, CYP11A1, 3β‐HSD, and 17β‐HSD in TM3 cells following treatment with AAPH. AAPH treatment reduced the expression levels of these proteins to varying degrees compared to the control group. Notably, the expression levels of 3β‐HSD and 17β‐HSD proteins exhibited significant differences compared to the control group (*p* < .05). However, the intervention of the three dipeptides increased the expression levels of all four proteins to varying degrees. Remarkably, the VR‐treated cells showed the most significant increase in the expression levels of these proteins. Furthermore, a strict dose dependence was observed, with higher doses of VR treatment leading to significant differences in the expression levels of all four proteins in the cells compared to the AAPH group (*p* < .05).

**FIGURE 9:**
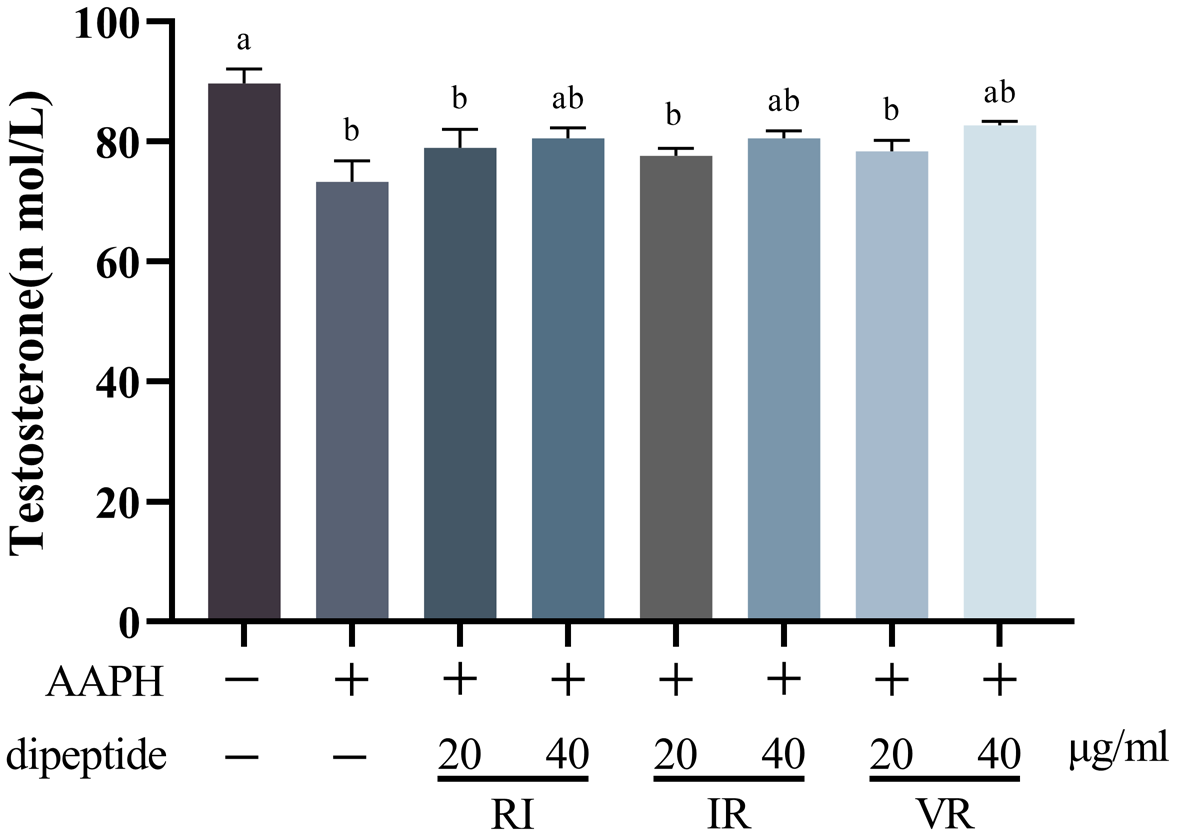
Testosterone content in cell culture supernatants, *n* = 3, different letters indicate significant differences.

Figure 9 presents the results of the testosterone assay conducted on the cell culture supernatant. Treatment with AAPH led to a significant decrease in testosterone secretion by the cells (*p* < .05). However, the three dipeptides exhibited varying degrees of reversal of this decrease, although no significant difference was observed compared to the AAPH group (*p* > .05). Furthermore, the increase in testosterone levels was observed to be correlated with the dose of dipeptides, suggesting a dose‐dependent effect on testosterone secretion.

### 3.7 ACE inhibitory activity of dipeptides

**FIGURE 10:**
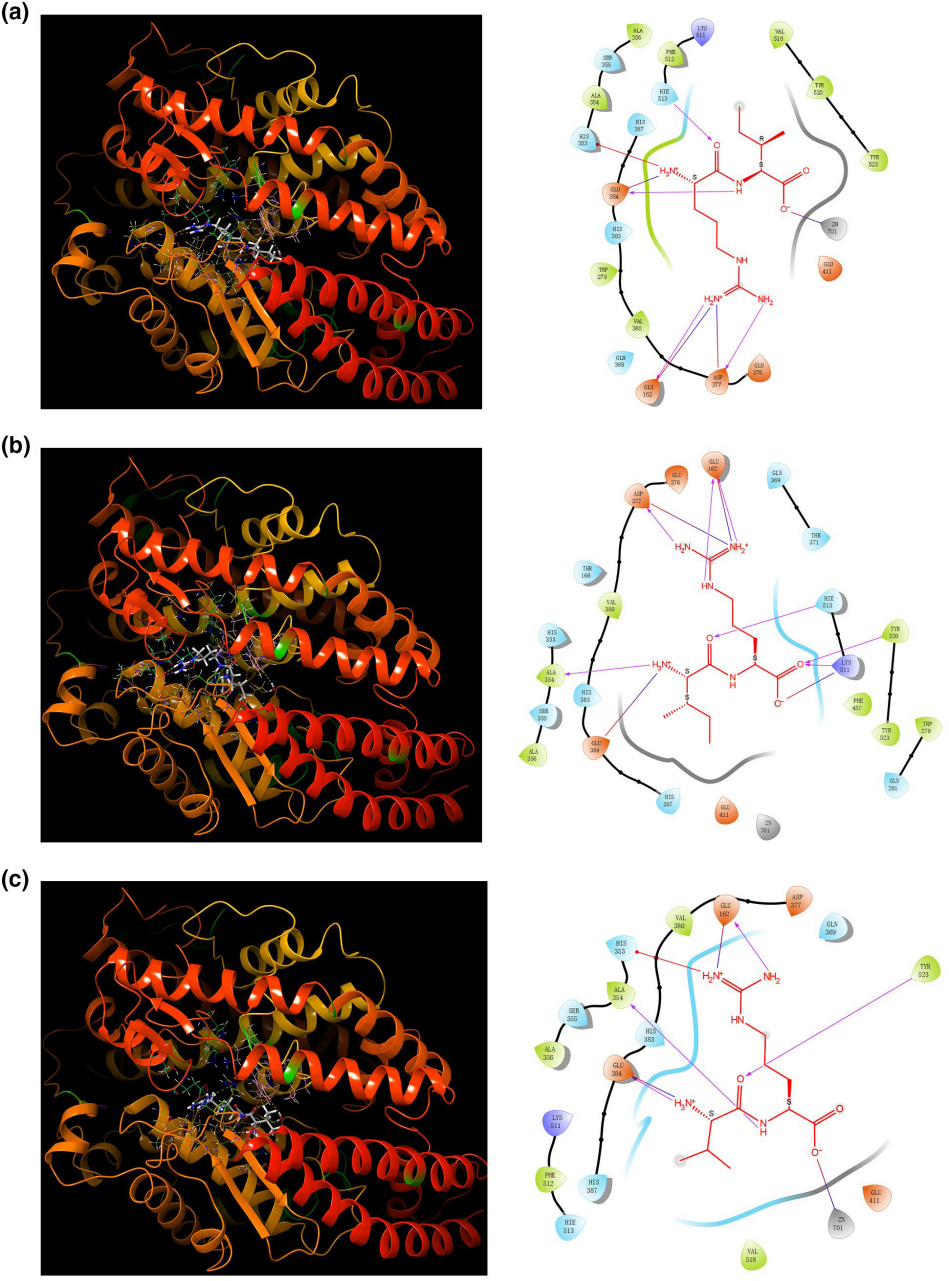
Docked interactions of RI (a), IR (b), and VR (c) with ACE (PDB: 1O86). Various colors are used to show the interactions with residues. The three‐dimensional structure of the peptide–protein complex is shown on the left, and the two‐dimensional interaction of the amino acid residues of the peptide and protein is shown on the right.

Figure 10 presents the results of the molecular docking analysis of the three dipeptides (RI, IR, and VR) with the ACE protein. The docking scores for dipeptides RI, IR, and VR with ACE were −7.234, −5.855, and −7.836, respectively. Dipeptide RI formed four hydrogen bonds with Glu162, Asp377, Glu384, and Hie513, a pi‐cation bond with His353, three salt bridges with Glu162, Asp377, and Glu384, and interacted with Zn701 (Figure 10a). Dipeptide IR interacted with ACE through seven hydrogen bonds with Glu162, Ala354, Asp377, Lys511, Hie513, and Tyr520, and formed four salt bridges with Glu162, Asp377, Glu384, and Lys511 (Figure 10b). Dipeptide VR formed four hydrogen bonds with Glu162, Ala354, Glu384, and Tyr523 of ACE, a pi‐cation bond with His353, two salt bridges with Glu162 and Glu384, and also exhibited interactions with Zn701 (Figure 10c). Most of the aforementioned amino acid residues are located in the active pocket of the ACE protein, indicating potential interactions between the dipeptides and the active site of ACE.

## 4 DISCUSSION

TM3 cells are a type of mouse testicular Leydig cells that serve as an in vitro model for investigating the effects of chemicals on testosterone biosynthesis (Bara & Kaul, 2018). This study showed that intracellular ROS levels increased significantly after treating TM3 cells with AAPH at a concentration of 600 μmol/L for 24 h. The excess ROS activated the NRF‐2 pathway, thereby upregulating the protein expression levels or enzyme activities of HO‐1, CAT, SOD, GST, and GSH‐Px downstream of NRF‐2. In addition, excess ROS activated the JNK pathway, leading to increased apoptosis. Oxidative stress inhibits the cell's ability to synthesize testosterone, caused at least by reduced expression levels of critical enzymes in the testosterone synthesis pathway. Intervention with the dipeptides RI, IR, and VR resulted in varying degrees of reduction in NRF‐2 and HO‐1 protein expression levels and a significant reduction in intracellular ROS levels. The activity of antioxidant enzymes regulated by NRF2 was decreased significantly, and JNK signaling was inhibited, indicating that cellular oxidative stress was alleviated. At the same time, the expression levels of critical enzymes in the testosterone synthesis pathway are increased, increasing testosterone synthesis.

Oxidative stress occurs when the level of ROS in cells exceeds the protective capacity of the body's antioxidant mechanisms (Aitken, 2020). NRF‐2 is a crucial regulator of cellular resistance to oxidative stress. In oxidative stress conditions, NRF‐2 is no longer degraded by ubiquitination but translocated to the nucleus to regulate the expression of associated protective genes, which are reduced when ROS production is inhibited (Guan et al., 2019). HO‐1 is widely used as a marker of oxidative stress and is an oxidative stress‐sensitive response protein regulated by NRF‐2 (Min et al., 2011; Tian et al., 2018). Activation of NRF‐2 also leads to upregulation of the expression of several antioxidant enzymes (Guan et al., 2019), mainly including superoxide dismutase (SOD), catalase (CAT), glutathione peroxidase (GSH‐Px), and glutathione sulfhydryl transferase (GST) (Baskaran et al., 2021; Song et al., 2018).

In the early stages of ROS overproduction, the activity of antioxidant enzymes in cells increases as a defense mechanism against ROS‐induced oxidative damage (Huchzermeyer et al., 2022; Vives‐Bauza et al., 2006). Among these, SOD catalyzes the conversion of superoxide radicals to hydrogen peroxide, CAT converts hydrogen peroxide to water; GSH‐Px reduces toxic peroxides to non‐toxic hydroxyl compounds, and GST plays a vital role in the detoxification of almost all electrophilic poisonous compounds (Hu et al., 2019). JNK proteins are members of the mitogen‐activated protein kinase family, and JNK transduces oxidative stress signals caused by ROS accumulation and induces apoptosis through phosphorylation activation (Kalender et al., 2010; Li, Zhou, Xu, et al., 2020).

Typically, the mechanisms by which bioactive peptides scavenge ROS include 1. neutralizing the toxicity of ROS by providing electrons or hydrogen for reduction reactions with ROS or forming stable complexes with ROS; 2. indirectly scavenging ROS by regulating the expression or activity of intracellular antioxidant enzymes and enhancing the antioxidant defense system of cells; and 3. reducing ROS production or precursors by affecting intracellular signaling pathways. The antioxidant activity of peptides is related to their amino acid composition and molecular weight; the smaller the peptide, the easier it is to reach the site of action in the cell and has more significant antioxidant activity (Mardani et al., 2023; Nwachukwu & Aluko, 2019); the order of the amino acids affects the highest occupied orbital energy and the active site of the peptide, thus affecting the antioxidant activity (Liu et al., 2022). Dipeptides have more significant antioxidant activity than the amino acids that make up their peptides, and dipeptides' residues can be absorbed more rapidly than free amino acids (Luo et al., 2013).

Hydrophobic amino acids at the *N*‐terminus are considered one of the characteristics of antioxidant peptides (Zou et al., 2016). Hydrophobic amino acids can easily cross the cell membrane's lipid bilayer, destroying intracellular reactive oxygen species because fatty acid radicals are hydrophobic and tend to bind to hydrophobic amino acid residues (Li & Yu, 2015). Due to the presence of imidazole groups that can act as proton donors, the hydrophobic amino acids leucine (I) and valine (V) are considered to have intense radical scavenging activity, especially in enzyme‐catalyzed reactions. However, too many hydrophobic amino acids reduce the solubility of the peptide and its antioxidant capacity (Xiang et al., 2023). The hydrophilic amino acids at the *C*‐terminus make it easier for the peptide to provide hydrogen atoms for bursting free radicals (Li et al., 2011). It has been shown that the high antioxidant activity of peptides may be related to the presence of arginine (R) at the *C*‐terminus and that the amino group in the R side chain may provide hydrogen for free radicals (Sae‐Leaw et al., 2017). Therefore, we presume that the R residues in the dipeptide sequence can react with ROS by providing electrons or protons to neutralize ROS. The guanidine group of R may oxidize with the hydroxyl radical (‐OH) to produce nitric oxide and carbon monoxide, thereby reducing the ROS. The guanidine group may also undergo a reduction reaction with H_2_O_2_ to produce water and nitrous acid. The guanidine group may also undergo elimination reactions with superoxide anion radicals (O2·‐) to produce nitric oxide and carbon dioxide.

Angiotensin‐converting enzyme (ACE) converts angiotensin I into angiotensin II. Angiotensin II can stimulate intracellular NADPH oxidase, producing reactive oxygen species (ROS). ACE inhibitors prevent the hydrolysis of angiotensin I by ACE, achieved through the formation of hydrogen and hydrophobic bonds that interact with multiple amino acid residues at the ACE active site. It has been demonstrated that the presence of the positively charged amino acid R at the C‐terminus of the peptide sequence can significantly influence the ACE inhibitory activity of the peptide. Additionally, V in the peptide sequence has been found to enhance the ACE inhibitory activity of the peptide (Liu et al., 2021). The dipeptides VR and IR have been reported to exhibit inhibitory activity against ACE (Ko et al., 2017; Liu et al., 2021). Molecular docking studies have revealed that the three dipeptides (VR, IR, and RI) can interact with multiple amino acid residues at the ACE active site by forming hydrogen bonds. This interaction prevents the hydrolysis of angiotensin I by ACE, thereby reducing the production of ROS.

The dipeptides IR and VR possess characteristics that align with antioxidant peptides, featuring hydrophobic amino acids at the *N*‐terminus and R at the C‐terminus. Furthermore, R has been shown to affect testosterone synthesis positively (Wang et al., 2002). These features may contribute to their antioxidant activity and ability to promote testosterone synthesis. On the other hand, although RI does not exhibit the typical profile of hydrophobic amino acids at the *N*‐terminus, it demonstrates significantly higher molecular docking scores with ACE proteins compared to IR. This difference in molecular docking scores may be attributed to RI's distinct amino acid sequences. The specific sequence and arrangement of amino acids in RI likely play a crucial role in its interactions with ACE proteins and antioxidant properties.

This study reports on three oyster‐derived dipeptides with antioxidant activity: RI, IR, and VR. The findings of this study provide evidence that the dipeptides investigated have the potential to enhance testosterone synthesis in TM3 cells. The results indicate that these dipeptides can effectively alleviate oxidative stress, increasing the expression of critical enzymes in the testosterone synthesis pathway. By mitigating oxidative stress, these dipeptides create a favorable cellular environment for testosterone synthesis, promoting its production. These findings highlight the potential of these dipeptides as promising agents for modulating testosterone synthesis through the regulation of oxidative stress in TM3 cells. In addition, these three dipeptides may alleviate the oxidative stress state of cells by directly scavenging excess ROS from cells or by reducing ROS production rather than increasing the activity of antioxidant enzymes in cells.

Oyster peptides are used mainly in food, pharmaceutical, and cosmetics. With the increasing awareness and demand for healthy and functional foods, oyster peptides are expected to play an important role in the food industry as a high‐value‐added marine biological resource. Factors such as the cost of raw materials, the preparation process, and the purification method determine the economic viability of oyster peptides. At present, the preparation process of oyster peptides mainly adopts enzymatic hydrolysis. Still, there are problems in enzyme selection, optimization of hydrolysis conditions, and product separation and purification, resulting in the high production cost of oyster peptides. Therefore, further research is needed to improve the preparation process of oyster peptides, increase their yield and quality, and reduce their cost, to improve their market competitiveness and economic benefits. In conclusion, the oyster peptide is a marine biological resource with great potential for development and market prospects, and it is worth further strengthening research and promoting its application.

## AUTHOR CONTRIBUTIONS

**Yongqiu Yan:** Conceptualization (equal); formal analysis (equal); investigation (equal); methodology (equal); supervision (equal); validation (equal); writing – original draft (equal). **Mingliang Li:** Conceptualization (equal); formal analysis (equal); investigation (equal); methodology (equal); supervision (equal); validation (equal); writing – original draft (equal). **Ying Wei:** Conceptualization (equal); funding acquisition (equal); supervision (equal); writing – review and editing (equal). **Fuhuai Jia:** Supervision (equal). **Yanying Zheng:** Funding acquisition (equal).

## FUNDING INFORMATION

This research was financially supported by the Key Project of Public Welfare Science and Technology Program of Ningbo (Grant No. 2021S001) and the Key Project of Science and Technology Innovation 2025 Foundation of Ningbo (Grant No. 2019B10060).

## CONFLICT OF INTEREST STATEMENT

The authors declare no conflict of interest.

## Data Availability

The data that support the findings of this study are available on request from the corresponding author. The data are not publicly available due to privacy or ethical restrictions.
